# Attitudes toward organ donation among university students in the United Arab Emirates: a cross-sectional survey

**DOI:** 10.3389/fpubh.2025.1551380

**Published:** 2025-04-16

**Authors:** Fayez Alshamsi, Ghada S. M. Al-Bluwi, Ricard Valero, Omran Bakoush

**Affiliations:** ^1^Department of Internal Medicine, College of Medicine and Health Sciences, United Arab Emirates University, Al Ain, United Arab Emirates; ^2^Department of Anesthesiology, Hospital Clínic de Barcelona, Universitat de Barcelona, Barcelona, Spain; ^3^Institut d’Investigacions Biomèdiques August Pi i Sunyer (IDIBAPS), Barcelona, Spain; ^4^Centro de Investigación Biomédica en Red Salud Mental (CIBERSAM), Madrid, Spain; ^5^Donation and Transplantation Institute, Barcelona, Spain

**Keywords:** Organ donation (topic area), public awareness, organ transplant, predictors, attitudes

## Abstract

**Introduction:**

Organ transplantation is a vital treatment for end-stage organ failure. However, the shortage of available organs remains a significant challenge worldwide. This study aimed to explore university students’ willingness to donate organs, including their views on brain death, in the United Arab Emirates (UAE).

**Methods:**

The study is cross-sectional survey on the respondents’ knowledge of, attitudes toward, and perceived barriers to and facilitators of organ donation. A total of 521 students completed the survey. Chi-square tests and logistic regression models were used to identify the factors associated with their willingness to donate their organs after death.

**Results:**

Most of the respondents (69%) were willing to donate their organs after death, and 79% were willing to donate their organs to a loved one during their lifetime. However, only 42.8% accepted brain death as equivalent to death. The most reported reasons behind the respondents’ willingness to donate organs were the belief that it is something everyone should do (adjusted odds ratio [aOR]: 4.68) and a responsibility to help loved ones (aOR: 2.63). Meanwhile, the significant barriers to organ donation included a preference for whole-body burial (aOR: 0.079), religious objections (aOR: 0.195), and family objections (aOR: 0.326).

**Discussion:**

University students in the UAE show a positive attitude toward organ donation. However, significant barriers, including family and religious objections, remain to be addressed. Increasing public awareness about brain death and establishing mechanisms for securing family consent in advance are crucial steps for the successful implementation of a deceased organ donation program in the UAE.

## Introduction

Organ transplantation is vital for end-stage organ failure, but rising demand has caused an organ shortage and growing waiting lists (1). In 2021, the Global Observatory on Donation and Transplantation reported organ availability at less than 10% of global demand, with most donations from deceased donors (2). In the UAE, deceased organ donation remains low despite an increasing number of patients awaiting kidney transplants (2–4).

Given the severe shortage of organs for donation in the country, a federal decree (5/2016) has been issued for the regulation of human organ transplantation. In 2017, the Ministry of Health and Prevention issued the first decree, defining the diagnostic criteria, rules, and procedures regulating the determination of death based on neurological criteria and subsequently updated it in 2022 (5). A recent systematic review reported that in the Middle East, up to half of the studied population were willing to donate their organs. However, the respondents’ knowledge regarding organ donation and their willingness to donate organs varied widely between studies (6). Another recent survey of residents in the UAE found that only 36% of the participants were aware that organ donation after death is allowed legally in UAE (7). Furthermore, there is limited information regarding the barriers and facilitators of organ donation among UAE residents.

As in most Arab and Eastern countries, health-related decisions in the UAE are shared family affairs; in many instances, family members provide social and supportive care for their sick family member (8). Moreover, people’s ideas and beliefs regarding illness; disease treatment; and the holiness of the human body, life, and death affect their decision-making and consent regarding donation after death (9–11).

In Eastern culture, death is an ominous matter; previous studies have highlighted that the fear of dehumanizing the body’s dignity, and the fear of the unknown prevents people from signing up for organ donation (11, 12). They also highlighted the importance of obtaining family members’ approval in signing up for organ donation. Moreover, a higher level of education and social media exposure could increase awareness and the willingness to donate organs (11, 13). Individuals with higher levels of education are more likely to perceive organ donation as a humane and noble act and are thus more likely to sign up for organ donation after death (14–19). Since the Federal Decree-Law No. 5 of 2016 on Regulation of Human Organs and Tissue Transplantation allowing and regulating organ transplantation between live and deceased donors was implemented in 2016, more than 110 deceased patients in the UAE have donated their organs. However, most of the donors were non-citizens and therefore number of donors among citizens remains low (2–4). Therefore, this study aimed to explore the willingness of university students in the UAE to donate their organs after death, including their views on brain death. We hypothesized that inadequate knowledge about donations processes, death by neurological criteria and unfavorable attitudes toward deceased donation may explain the low donation rates. Understanding the contextual factors that affect the deceased organ donation program will help in tailoring the awareness program to address family concerns about deceased organ donation. It will also guide policymakers in developing a contextual holistic approach to decrease the wastage of potential organs due to delay in consent and the counseling process.

## Materials and methods

**Figure 1:**
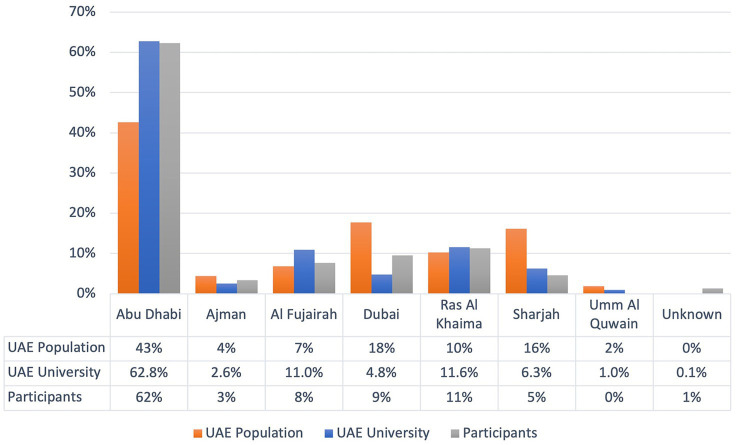
Distribution of the population, UAE University students, and study respondents across the UAE.

The UAE is a confederation of seven emirates. The population is multiethnic, with the majority (85%) being expatriate temporary workers (20). This study was conducted at UAE University because it is a federal public university that enrolls more than 13,000 students from all seven emirates (Figure 1). The study was approved by the Social Sciences Research Ethics Committee of UAE University (ERS_2018_5842).

A self-administered questionnaire was designed based on previous studies (21–23) and comprised three parts: (1) participants’ demographics and socioeconomic status; (2) their knowledge, attitude, and willingness to donate organs after brain death; and (3) the perceived barriers and facilitators for deceased organ donation.

The questionnaire was reviewed by three experts to assess the face validity of the survey items and to provide feedback regarding the clarity and conciseness of the questions. The ambiguous issues were corrected and then a pilot test was conducted using a test group of university students (*n* = 10). No problems were identified in the pilot study; therefore, no further changes were made to the questionnaire. The questionnaire was then posted online using Research Electronic Data Capture (REDCap), a secure web-based application that allows respondents to answer questions while preventing duplicate responses. Additionally, REDCap includes features that ensure data integrity and participant confidentiality, such as audit trails, real-time data validation, encryption, and customizable user permissions (24).

Next, REDCap was used to send invitations to students’ university e-mails on March 3, 2019. Three weekly reminders were sent to nonresponders who received the invitations. The responses were collected during the four-week period from March 3, 2019 to April 4, 2019. The first page of the survey contained information about the study’s rationale and statements ensuring respondents’ confidentiality. The participants were given the choice to decline or consent to accept the participation and proceeded to answer the survey questions. The survey questions allowed for no response as an option for every question, and the participants could stop participation at any time.

It was estimated that a sample size of 385 would provide correct information regarding respondents’ willingness to donate organs within a 5% margin of error at a 95% confidence level. The sample size was calculated using the following formula: *N* = Z^2^pq/d^2^, assuming a population proportion of 0.5 and an unlimited population size^1^.

The participants’ demographic characteristics were summarized using descriptive statistics. Continuous variables were summarized as means with standard deviations, whereas categorical variables were summarized as counts and percentages. The association between responses and variables related to participants’ demographics, knowledge, attitude, and willingness to donate organs after brain death and perceived barriers and facilitators for deceased organ donation was examined. A chi-squared test for comparing proportions was used to test the null hypothesis (i.e., there was no difference in the characteristics of participants who were willing to be, not willing to be, and unsure about being organ donors). Univariate and multivariable logistic regression models were constructed to determine the association of participants’ characteristics that could affect the decision to donate an organ. The statistical difference of the categorical variables was assessed using chi-square tests. Logistic regression analysis was then used to explore main factors affecting willingness to donate organs. Demographic variables that had a *p*-value <0.05 following the chi-squared test, and those that had a known association with organ donation hesitancy were included in a multivariate regression model. Odds ratios (ORs) with 95% confidence intervals (CIs) were calculated. A complete case analysis approach was applied in the regression analysis, where only participants with complete data for the variables included in each model were analyzed. Since all covariates are fully observed, there is no loss of information regarding the predictors. Statistical analyses were performed using the Statistical Package for the Social Sciences (SPSS V.26.0, IBM, Chicago, Illinois, USA).

## Results

### Characteristics of the study respondents

Of the 800 students who received the survey, 521 agreed to participate and completed the survey (response rate: 65.1%). The demographic characteristics of the study participants and their geographical distribution by emirate are shown in Table 1. The cohort of 521 students was found to be representative of the population distribution in the seven emirates (Table 1, Figure 1).

**Table 1:** Respondents’ demographic characteristics.

Key descriptive characteristics	*N* = 521	%
Gender		
Male	57	10.9
Female	459	88.1
Not declared	5	1.0
Age		
<25	486	93.3
≥25	29	5.6
Not declared	6	1.2
Nationality		
UAE	398	76.4
Expat	77	14.8
Not declared	46	8.8
Economic status		
Low	201	38.6
High	126	24.2
Not declared	194	37.2
Education		
Health sciences	25	4.8
Humanities	496	95.2
Emirate		
Abu Dhabi	242	46.4
North Emirates	142	27.3
Expat	77	14.8
Not declared	60	11.5
Views on organ donation		
Support	437	83.9
Oppose	17	3.3
Not sure	67	12.9
Knows someone who needs an organ transplant		
Yes	185	35.5
No	336	64.5

Most respondents were Emirati nationals (~76%) and female (~88%), with a nonmedical background (humanities: 496 [95.2%]). Most of the respondents supported organ donation as a medical treatment (84%). Approximately one-third of the respondents knew someone who needed an organ transplant or already had an organ transplant (Table 1).

Approximately 33% of the respondents were not aware or unsure about the existence of an organ donation program in the UAE (Table 2). Others had learned about the UAE organ donation program primarily through media and awareness campaigns. A few respondents had learned about the program in schools.

**Table 2:** Respondents’ willingness to donate organs.

Key descriptive characteristics	*N* = 521	%
Willingness to donate while alive		
Agree	420	80.6
Disagree	31	6.0
Not sure	70	13.4
Willingness to donate after death		
Agree	358	68.7
Disagree	73	14.0
Not sure	90	17.3
Willing to consent for donating family members’ organs		
Yes	175	33.6
No	84	16.1
Not sure	262	50.3
Discussed organ donation with others		
With a family member	194	37.2
Others	95	18.2
No/not sure	232	44.5
Informed close relatives regarding their wish to donate organs after death		
Yes	369	70.8
No	54	10.4
Not sure	98	18.8
Familiarity with brain death		
Yes	436	83.7
No	85	16.3
Accept brain death as death		
Yes	219	42.0
No	129	24.8
Not sure	173	33.2
Aware of the UAE organ registry		
Yes	267	51.2
No	254	48.8
Willing to register		
Yes	265	50.9
No	63	12.1
Not sure	193	37.0
Consent for donation of a deceased relative’s organs		
Agree	160	30.7
Agree if consented	210	40.3
Disagree	53	10.2
Not sure	98	18.8

### Willingness and attitude toward organ donation

Most respondents (79%) were willing to donate their organs to a loved one during their lifetime (Table 2). In addition, most (69%) of the respondents were willing to donate their organs after death. However, 50% of respondents hesitated to consent for donating a family member’s organ after their death. Approximately 55% of the respondents had discussed the topic of organ donation, most of the time, they discussed this issue with a family member (Table 2). They discussed being a donor and their opinions on organ donation (Table 2). When the topic was not discussed, it was usually because it never came up during conversations (Table 2).

The characteristics of respondents who were willing to donate their organs after death are shown in Table 3. The willingness for organ donation was significantly higher among those who were willing to donate their organs while alive, those who registered as organ donors, those who consented for the donation of a deceased relative’s organs, and those who had expressed their wishes to donate after death.

**Table 3:** Factors contributing to willingness to donate organs after death (logistic regression).

Willingness to donate after death (yes/no)					
Variable	B	SE	Sig	OR	95% CI
Support organ donation in principle	2.594	0.287	<0.001	13.377	7.628–23.458
Willing to donate organs to loved ones while alive	1.735	0.236	<0.001	5.668	3.569–9.001
Consent for donation of a deceased relative’s organs (Yes)	2.247	0.318	<0.001	9.455	5.065–17.650
Familiar with the term brain death	0.502	0.247	0.042	1.652	1.018–2.681
Accept that brain death means death	0.615	0.202	0.002	1.849	1.244–2.749
Discuss organ donation with others	0.499	0.193	0.010	1.648	1.128–2.406
Discuss being an organ donor	1.278	0.224	<0.001	3.591	2.313–5.574
Aware of the organ donation register	0.479	0.194	0.013	1.614	1.104–2.360
Willing to register as an organ donor	3.074	0.302	<0.001	21.638	11.973–39.106
Important to inform others about my wish to donate	1.488	0.208	<0.001	4.429	2.946–6.660
If a family member is pronounced dead: Agree to donate their organs	0.828	0.204	<0.001	2.289	1.533–3.417

B, unstandardized coefficient; SE, standard error; Sig, significant; OR, odds ratio; CI, confidence interval.

### Perception and attitude toward brain death

Most of the respondents were familiar with the term brain death (83.7%). However, only 42.8% of respondents accepted brain death as equivalent to death (Table 2), with the main reason for rejecting brain death as death being that the individual still had a heartbeat.

### Willingness to register for organ donation

**Figure 2:**
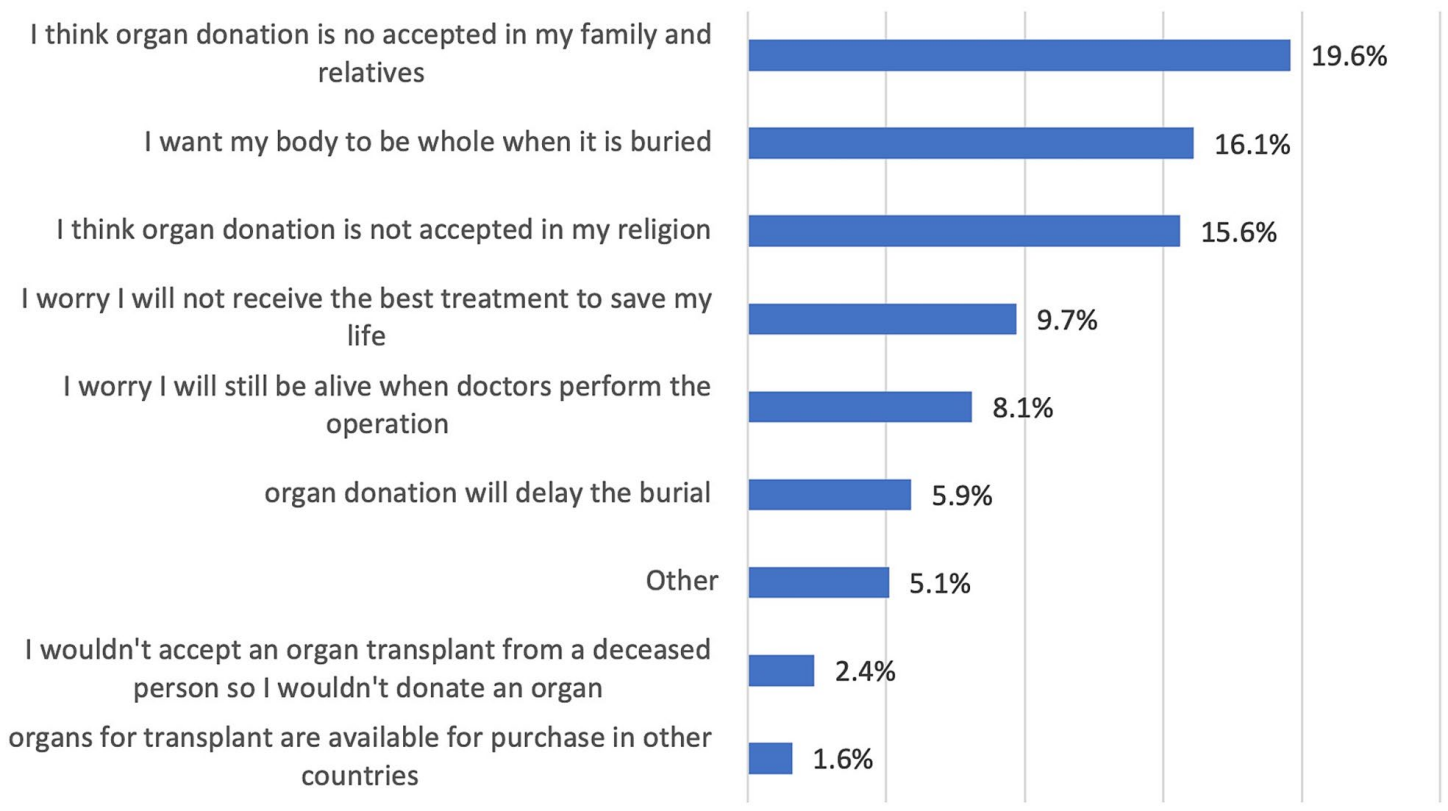
Reasons for not donating organs after death.

Approximately 51% of respondents were aware of the existence of the UAE register for organ donation and were willing to register for organ donation after death (Table 2). However, 19.6% mentioned that they would consider their family members’ opinions before registering as organ donors (Figure 2).

**Figure 3:**
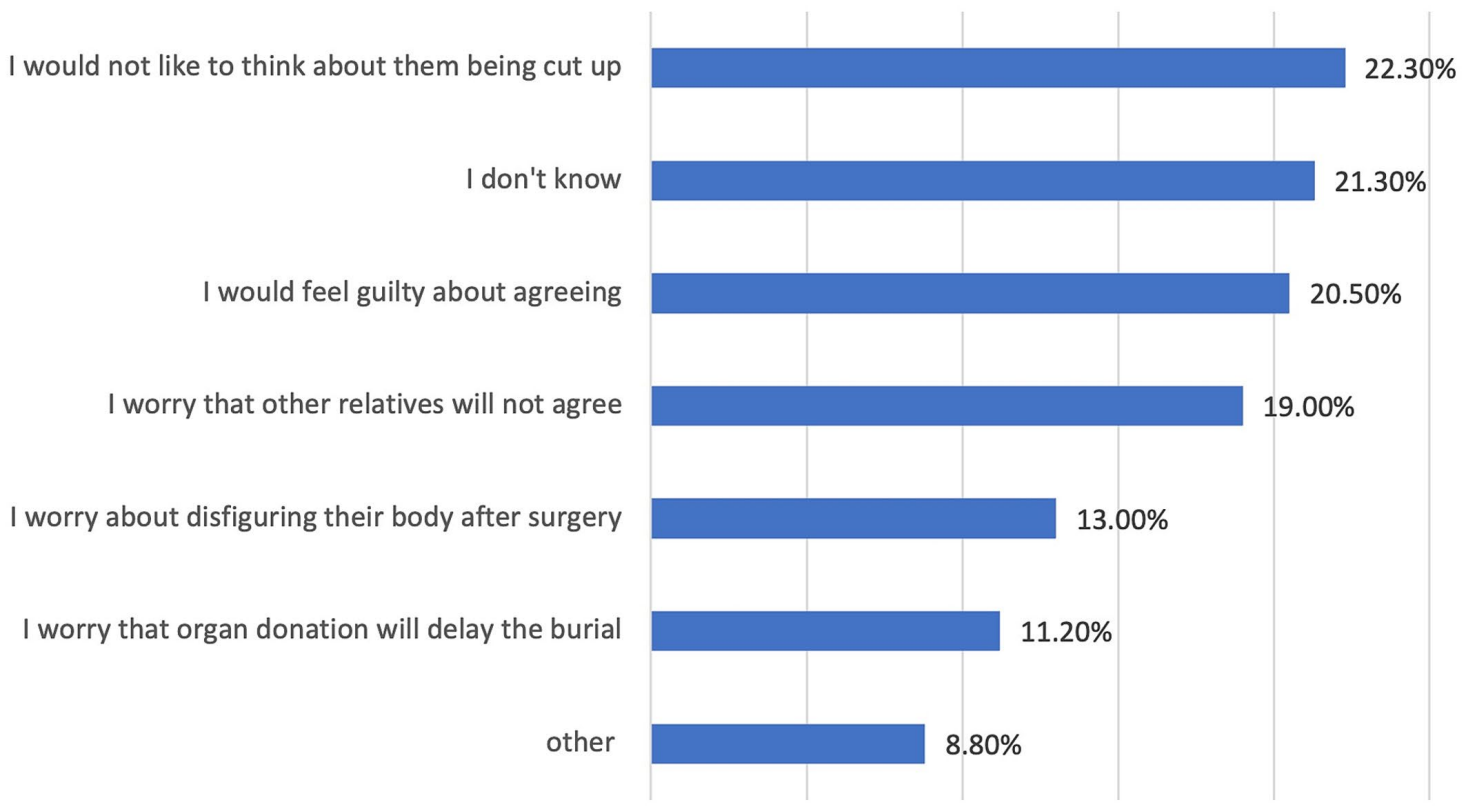
Reasons for refusing consent to donate the organs of relatives after their death.

Approximately 71% of respondents agreed that it is important to inform people close to them about their wish to donate organs after death, and many would agree for a family member to donate their organs only if they had expressed their wishes or consent in writing to a register (Table 2). Because of personal beliefs or sociocultural reasons, 29% of respondents either disagreed or were hesitant to agree to donate the organs of a family member (Figure 3).

### Perceived facilitators and barriers toward organ donation

Almost all respondents perceived organ donation as a good act that should be promoted (82.7%) and could save someone’s life (93.3%). Nevertheless, many respondents were unlikely to register as an organ donor even if their families had no objection (26.3%). Many respondents did not believe that organ donation would be rewarded by God (38.6%), and few respondents believed that organ donation is permitted in Islam (23.4%) and would be accepted in the UAE society (20.2%).

### Predictors toward organ donation while alive

The most reported reasons of respondents for their willingness to donate organs while alive were altruism and feeling obligated to help family members (Table 4). The most commonly selected responses were that the respondents believed that organ donation is something that everyone should do (adjusted OR [aOR]: 4.68; 95% CI: 1.635–13.413); that it is a responsibility to help a loved one (aOR: 2.63; 95% CI: 1.654–4.196); that it should be done as respondents would like to accept an organ transplant themselves (aOR: 2.45; 95% CI: 1.385, 4.353); and that it will save others’ lives (aOR: 1.63; 95% CI: 1.024, 2.593).

**Table 4:** Perceived reasons for willingness to donate organs while alive.

	B	SE	Sig	OR	95% CI
A: Reasons for willingness to donate organs while alive (multivariate)					
I feel responsible for helping my loved ones	0.969	0.238	<0.001	2.634	1.654–4.196
I would accept an organ transplant, so I should be prepared to donate one	0.898	0.292	0.002	2.455	1.385–4.353
It would improve and save the lives of others	0.488	0.237	0.039	1.629	1.024–2.593
It is something that everyone should do	1.544	0.537	0.004	4.683	1.635–13.413
I feel obliged to donate if asked	1.421	0.764	0.063	4.142	0.927–18.505
B: Reasons for unwillingness to donate organs while alive (multivariate)					
I would not accept an organ transplant myself, so I need not donate one	−1.859	0.637	0.004	0.156	0.045–0.543
I am afraid of the operation	−0.851	0.274	0.002	0.427	0.250–0.730
I am afraid I will become sick after losing my organ	−1.393	0.278	<0.001	0.248	0.144–0.428
It is not possible to perform donation from a living organ donor in the UAE	−1.049	0.447	0.019	0.350	0.146–0.842

B, unstandardized coefficient; SE, standard error; Sig, significant; OR, odds ratio; CI, confidence interval.

Respondents who were hesitant to donate organs frequently cited fear of the operation or of losing an organ. The reported reasons for being unwilling to donate organs included the participants attitude against accepting deceased organ transplantation (aOR: 0.156; 95% CI: 0.045–0.543), fear of sickness (OR: 0.248; 95% CI: 0.144–0.428), unavailability of donating organs while living in the UAE (aOR: 0.35; 95% CI: 0.146–0.842), and fear of the operation (OR: 0.43; 95% CI: 0.250–0.730).

### Predictors toward organ donation after death

The characteristics of respondents who were willing to donate organs after death are shown in Table 3. The reported reasons for the willingness to donate organs after death included altruism and the obligation to help family members. The respondents who were hesitant to donate their organs after death were primarily hesitant because of sociocultural and religious beliefs (Table 5). The odds to donate organs after death decreased by 92.1% (aOR: 0.079; 95% CI: 0.041–0.152), 80.5% (aOR: 0.195; 95% CI: 0.102–0.373), and 67.4% (aOR: 0.326; 95% CI: 0.187–0.568) for participants who wanted their whole body to be buried after death, those who had religious objections, and those who had family objections, respectively.

**Table 5:** Perceived reasons for willingness to donate organs after death.

	B	SE	Sig	OR	95% CI
A: Reasons for willingness to donate organs after death (multivariate)					
I would accept an organ transplant, so I should be prepared to donate one	1.613	0.391	<0.001	5.019	2.332–10.800
I feel responsible for helping people in need	1.318	0.299	<0.001	3.736	2.081–6.708
It would improve and save the lives of others	1.007	0.245	<0.001	2.737	1.692–4.427
It is something that everyone should do	1.281	0.429	0.003	3.599	1.553–8.337
It makes me feel good to know that I could be helping someone when I die	1.083	0.249	<0.001	2.954	1.814–4.812
B: Reasons for unwillingness to donate organs after death (multivariate)					
I want my body to be whole when it is buried	−2.537	0.333	<0.001	0.079	0.041–0.152
I think organ donation is not accepted by my family and relatives	−1.120	0.283	<0.001	0.326	0.187–0.568
I think organ donation is not accepted in my religion	−1.635	0.331	<0.001	0.195	0.102–0.373
I would not accept an organ transplant from a deceased person, so I need not donate an organ	−1.576	0.928	0.089	0.207	0.034–1.026
I worry that I may still be alive when the doctors perform the operation	−0.764	0.403	0.058	0.466	0.212–1.026

B, unstandardized coefficient; SE, standard error; Sig, significant; OR, odds ratio; CI, confidence interval.

## Discussion

This study aimed to explore the perceived facilitators and barriers toward organ donation in the UAE. Although most participants had a positive attitude toward organ donation and were willing to donate their organs to a loved one while alive, they were less likely to donate after death. Commonly cited reasons for declining consent for organ donation included fear of family refusal, moral objections to taking human organs after death, concerns about body disfigurement, and religious beliefs. The study’s findings align with previous research conducted elsewhere, which highlights that religious beliefs and cultural reasons are significant barriers to the acceptance of deceased organ donation in the Middle East (6, 13, 25).

The present study included university students, who typically have higher levels of education and are more likely to be open to sociocultural changes (14). Our findings concur with a similar study involving university students in Turkey. In that study, willingness to donate organs among university students was associated with receiving education on the topic, previous discussions with family and friends, awareness of existing organ donation program, willingness to receive an organ if needed and being registered as organ donors (26). While many participants demonstrated knowledge of brain death, a significant portion did not equate brain death with actual death. Instead, most participants accepted death only when the heart and breathing ceased or when all body organs stopped functioning. The prevailing belief that death cannot be declared if the heart is still beating underscores the need for targeted educational efforts about what brain death truly means as an irreversible cessation of all brain activity and is legally recognized as death. The confusion induced by the link of brain death concept with organ donation is highlighted by our study findings of a paradoxical correlation between participants acceptance of brain death as equivalent to death and their willingness to donate after death (27). Therefore, dissociating brain death from organ donation discussions might be prudent for the success of organ donation program in this setting. Multifaceted outreach initiatives that clarify religious, clinical, and legal perspectives on death based on neurological criteria, could improve public understanding of brain death. Collaborating with religious leaders and cultural influencers to provide accurate information about organ donation may provide culturally sensitive support to medical teams approaching families for organ donation authorization (27).

Although many respondents were willing to donate their organs after death (69%), 50% of participants in our study expressed hesitation in consenting to donate a deceased relative’s organs. Family participation in decisions to donate organs after death is a norm in society, and their consent is often shaped by sociocultural and religious beliefs (8, 12, 14). This hesitation can create ethical dilemmas related to respecting the autonomy of the deceased, managing the emotional burden on families, and ensuring cultural sensitivity (27, 28). Additionally, it can lead to delays in decision-making, potentially affecting the success of organ transplantation efforts. Addressing this issue requires public awareness initiatives that encourage open discussions about organ donation within families and promote the documentation of individual donation preferences (29, 30).

Religious and sociocultural beliefs are commonly cited barriers for consenting for organ donation after death (31). Individuals who are willing to register as donors would seek the consent of family members and perhaps religious figures before registering as organ donors (32, 33). The Islamic perspective on organ donation and transplantation has been extensively reviewed; overall, the religion has a positive attitude toward this subject (34). However, this view might not be widely prevalent. For example, a survey of clerics in Turkey showed poor knowledge of and attitudes toward organ donation (35). Thus, it is important to address cultural issues during organ retrieval and provide information about the process of organ retrieval in a way that aligns with cultural needs (e.g., declaring death, not delaying the burial, and preserving the body after retrieval). These findings also emphasize the importance of educating the public regarding the process of organ retrieval and how healthcare professionals consider cultural needs (12). A recent systematic review with narrative synthesis exploring barriers and facilitators of deceased organ donation among Muslims living in various communities globally identified the following five themes as important: knowledge, willingness to donate, community influence, bodily influence and religious beliefs (36). Furthermore, it is important for the public to be informed that a person has died when an official declaration of brain death is made (33).

Participants who believed that organ donation may be rewarded by God were more likely to register as a donor, highlighting the impact of religious belief on attitude toward organ donation (11, 12). In addition, a mechanism is needed to establish an early interaction with family members of potential donors facilitate the family consenting process and facilitating successful organ donation process (37). Furthermore, 10% of respondents reported fear of not receiving appropriate treatment as a barrier for registering as an organ donor (Figure 2). Donor rights are among the priorities for policymakers in the UAE as in the updated and more detailed Federal Decree by Law No. (25) of 2023 Concerning Donation and Transplantation of Human Organs and Tissues (38).

The strengths of our study are as follows: First, the study fills a gap in the literature regarding public knowledge regarding organ donation in the UAE and the barriers and facilitators to organ donation in the country. Second, the study adheres to recognized standards for formulating self-administered questionnaires, improving the robustness of the findings (39). Third, the study’s sample represents a fair distribution of respondents over the UAE’s seven emirates, improving the generalizability of the findings. Notably, however, this study also has some limitations, including a small sample size; a closed sampling frame (students at UAE University), which may not be representative of society; and low representation of males (10.9%). Low response rate is a common concern for surveys which can affect the strength of findings and increase bias. In our study, the response rate of 65%, despite being low, is above the suggested rate of 45% for online survey (40). Several factors may explain the poor response rates of males compared to females including organ transplantation topic itself (41). For example, a survey on this topic done in Turkey, a country that shares similar social and cultural beliefs as ours, 84% of respondents were females (42). This suggests that topic might not be of interest to males. Additionally, research has indicated that females generally respond more frequently to online surveys compared to their male counterparts, possibly due to differences in engagement with academic research and survey participation (43, 44). Therefore, a mixed-method approach for data collection that allows free-text answers, qualitative in-depth interviews including family members, or focus group discussions, can provide valuable additional information.

The concept of brain death is still foreign among Arab and eastern societies. The low rate of acceptance of the diagnosis of brain death is related to the existing gap between medical knowledge, legality of brain death, religious views, and the public acceptance of diagnosis of the brain death. This complicated by the central influence of family in any heath care related decision-making process. The notion that an individual is dead, according to medical definition, while the heart is still beating causes a lot of turbulence between the patient’s family and the caring physician. To resolve this issue, the medical community can improve understanding through consistent, simplified language, dissociating brain death from organ donation, and recognizing the emotions tied to discussions of brain death. The educational effort should focus on to increase public overall awareness of brain death as a medical condition. Furthermore, there is a need to form clear practical guidelines related to the diagnosis of brain death and subsequently guidelines related to the withdrawal of artificial support in these patients.

In conclusion, the study findings shed light on the limited acceptance of brain death and the need to obtain family consent for deceased organ donation. The public’s sociocultural view of death needs to evolve for deceased organ donation rates to improve. A structured approach, including early, empathetic communication with families of potential deceased donors, is essential. This should provide clear information about brain death, address cultural and religious concerns, and offer necessary religious and culturally support to the family. Additionally, preemptive education and involvement of trusted community leaders can help to improve understanding of brain death concept as terminal death. Future research on the best policy strategies and best practices for obtaining consent for deceased organ donation is needed.

## Data Availability

The raw data supporting the conclusions of this article will be made available by the authors, without undue reservation.

## Footnotes

^1^ https://www.calculator.net/sample-size-calculator.html?type=1&cl=95&ci=5&pp=50&ps=&x=Calculate
