# Supplementary material for: Attitudes toward organ donation among university students in the United Arab Emirates: a cross-sectional survey
Source: Front Public Health. 2025 Apr 16;13:1551380. doi: 10.3389/fpubh.2025.1551380 (PMC12042666; doi:10.3389/fpubh.2025.1551380)
Supplement: Supplementary file 1 [file Data_Sheet_1.docx]

**Questionnaire**

**Knowledge and attitudes:**

1. Views on organ donation

Which of these statements best describe your views on organ donation as a medical treatment?

I strongly support organ donation in principle

I support organ donation in principle

I neither support nor oppose organ donation in principle

I oppose organ donation in principle

I strongly oppose organ donation in principle

I am not sure

1. Do you know anyone in need of an organ transplant (kidney, liver, heart, or lungs)?

Yes/no

1. Do you know anyone who has had an organ transplant (e.g., kidney transplant)?

Yes/no

**Organ donation while alive:**

1. I am willing to donate my organs to a loved one if needed.

Strongly agree

Agree

Neither agree nor disagree

Disagree

Strongly disagree

Not sure

1. I am willing to donate my organs to a loved one because

Please select all that apply.

I feel responsible for helping my loved ones

I would accept an organ transplant, so I should be prepared to donate one

People who are organ donors are viewed as virtuous

It is better to have an organ from someone you know

It would improve and save the lives of others

It is something that everyone should do

I feel obliged to donate if asked

I am NOT willing to donate my organs

If others, please write below:

1. I am NOT willing to donate my organs because

Please select all that apply.

I would not accept an organ transplant myself, so I need not donate one

I am afraid of the operation

I am afraid of losing my life

I am afraid I will become sick from losing my organ

It is not possible to perform a donation from a living organ donor in the UAE

I am concerned about the financial costs of the operation

I am willing to donate my organs

If others, please write below:

**Organ donation after death:**

1. I am willing to donate my organs after my death.

Strongly agree

Agree

Neither agree nor disagree

Disagree

Strongly disagree

Not sure

1. I want to donate my organs after death because:

Please select all that apply.

I would accept an organ transplant, so I should be prepared to donate one

People who are organ donors are viewed as virtuous

I feel responsible for helping people in need

It would improve and save the lives of others

It is something that everyone should do

It makes me feel good to know that I could be helping someone when I die

It is possible to perform organ donation after death in the UAE

I do NOT want to donate my organs after my death

Others; please write below:

1. I do NOT want to donate my organs after my death because:

Please select all that apply.

I worry I will not receive the best treatment to save my life

I want my body to be whole when it is buried

Organ donation will delay the burial

I think organ donation is not accepted by my family and relatives

I think organ donation is not accepted in my religion

I would not accept an organ transplant from a deceased person, so I need not donate an organ

I worry that I may still be alive when the doctors perform the operation

Organs for transplantation are available for purchase in other countries

I want to donate my organs after my death

If other, please write below:

**Consent for organ donation:**

1. I will agree for the organs of my family member to be donated after their death:

Yes

No

I do not know

1. The meaning of death is

Please select all that apply.

The heart is not beating, and there is no breathing

The brain stops working completely

I do not know

If other, please specify below:

1. Are you familiar with the term “brain death” (“الموت الدماغي”)?

Yes/no

1. I understand that a person is not alive anymore if they develop brain death.

Yes/no/not sure

1. I agree that a person is not alive anymore if they develop brain death because:

Please select all that apply.

It is my personal belief

Brain death is adopted in my religion

Brain death is adopted by the law in the UAE

Brain death is accepted in my society

If other, please specify below:

1. I DO NOT agree that a person is not alive anymore if they have brain death because:

Please select all that apply.

It is my personal belief

Brain death is against my religious beliefs

Brain death is not acceptable in my society

You cannot be dead if your heart is beating

I agree that a person is not alive anymore if they have brain death

Others; please specify below:

1. I agree to keep a dead person on artificial life support (e.g., breathing machine) for the purpose of organ donation.

Yes/no/I do not know

reset

**Barriers:**

1. I have discussed the topic of organ donation with others.

Yes/no/cannot remember

reset

1. I did NOT discuss organ donation with others because:

Please select all that apply.

I do not accept organ donation in principle

I am scared to talk about death

I am not sure about their opinion on organ donation

It never came up in our conversations

I have not thought about organ donation before

I do not know

I have discussed organ donation with others

If other, please specify below:

1. It is important to inform people close to me about my wish to donate my organs after my death.

Yes/no/I do not know

Below are some situations where people have had to make difficult decisions.

Please read through each situation and mention whether you think you would agree or not if you experienced that situation.

1. If a family member was diagnosed to be brain dead:

I would agree to donate their organs

I would not agree to donate their organs

I would agree only if they left a written wish to donate their organs

I would agree only if they had registered as an organ donor in the organ donor’s registry

I am not sure what I would do

reset

1. I refuse to donate the organs of a family member after their death because:

Please select all that apply.

I would not like to think about them being cut up

I worry about disfiguring their body after surgery

I worry that other relatives will not agree

I worry that organ donation will delay the burial

I would feel guilty about agreeing

I do not know

I agree to donate the organs of a family member after their death

Others; please specify below

1. I learned about organ donation in the UAE from

Please select all that apply

School or college curriculum

Media

Awareness campaigns

Not sure

I did not see or hear about organ donation

Others; please specify below:

1. I am aware that there is an organ donor registry in the UAE where people can indicate their wish to donate their organs after death.

Yes/no

1. I am willing to register as an organ donor.

Yes/no/not sure

1. To register as an organ donor, I will take the opinion of

Please select all that apply.

Family members

Healthcare professionals (e.g., doctors, nurses)

Religious person (shaikh)

Friends/colleagues

No one

Not sure

If other, please specify below:

1. Please answer the following questions:

Strongly agree Agree Neither agree nor disagree Disagree Strongly disagree

- Organ donation is a good thing and should be promoted
- Registering as an organ donor could save someone’s life
- Organ donation is an act that will be rewarded by God
- I am willing to register as an organ donor if my family would have no objections
- Organ donation will increase if social support is provided to the donor’s family
- I am concerned about the feelings of my family members while my organs are being taken
- Doctors will not offer me the best treatment to save my life if I was registered as an organ donor
- I do not trust the healthcare system in the UAE, and it is better to go abroad for organ transplantation

1. Please rate the factors below as to how likely they are to be potential barriers for your family to consent for organ donation:

Very likely Likely I do not know Unlikely Very unlikely

- After death, organ donation is forbidden (*haram*) in Islam
- After death, organ donation is not accepted in society
- After death, organ donation is illegal under UAE law
- The concept of brain death is not accepted by my family

1. In your opinion, what do you think can increase awareness of organ donation in the UAE?
2. In your opinion, what are the most important barriers to organ donation in the UAE?

Demographics:

1. Gender: female/male
2. Age:

<20/20–24/25–29/30–34/35–39/40–44/>44

1. Estimated monthly income of family (dirhams):

5000/5000–9999/10,000–19,999/20,000–29,999/30,000–39,999/40,000–49,999/>50,000/Prefer not to provide

1. Nationality:
2. College:

Business and economics/education/engineering/food and agriculture/humanities and social sciences/information technology/law/medicine and health sciences/science
